# Biophysical characterization of melanoma cell phenotype markers during metastatic progression

**DOI:** 10.1007/s00249-021-01514-8

**Published:** 2021-03-17

**Authors:** Anna Sobiepanek, Alessio Paone, Francesca Cutruzzolà, Tomasz Kobiela

**Affiliations:** 1 Laboratory of Biomolecular Interactions Studies, Chair of Drug and Cosmetics Biotechnology, Faculty of Chemistry, Warsaw University of Technology, Noakowskiego 3, 00-664 Warsaw, Poland; 2 Department of Biochemical Sciences “A. Rossi Fanelli”, Sapienza University of Rome, Rome, Italy

**Keywords:** Melanoma, Metastasis, Metabolism, Glycosylation, Label-free techniques

## Abstract

Melanoma is the most fatal form of skin cancer, with increasing prevalence worldwide. The most common melanoma genetic driver is mutation of the proto-oncogene serine/threonine kinase *BRAF*; thus, the inhibition of its MAP kinase pathway by specific inhibitors is a commonly applied therapy. However, many patients are resistant, or develop resistance to this type of monotherapy, and therefore combined therapies which target other signaling pathways through various molecular mechanisms are required. A possible strategy may involve targeting cellular energy metabolism, which has been recognized as crucial for cancer development and progression and which connects through glycolysis to cell surface glycan biosynthetic pathways. Protein glycosylation is a hallmark of more than 50% of the human proteome and it has been recognized that altered glycosylation occurs during the metastatic progression of melanoma cells which, in turn facilitates their migration. This review provides a description of recent advances in the search for factors able to remodel cell metabolism between glycolysis and oxidative phosphorylation, and of changes in specific markers and in the biophysical properties of cells during melanoma development from a nevus to metastasis. This development is accompanied by changes in the expression of surface glycans, with corresponding changes in ligand-receptor affinity, giving rise to structural features and viscoelastic parameters particularly well suited to study by label-free biophysical methods.

## Introduction

The most fatal skin-related cancer worldwide is melanoma, which develops as a result of mutation and uncontrolled proliferation of melanocytes, the skin cells producing the pigment melanin. Most melanoma cells retain the ability to produce melanin, thus they form pigmented (typically brown or black) melanomas. But in rare cases with disrupted melanin synthesis, amelanotic melanoma may occur. The primary site of melanoma development is the skin, but cases of the eye (e.g., uvea), meninges and various mucosal surfaces (e.g., oral mucosa) have also been reported. Many different clinical subtypes of melanoma have been described, among which the most common are: acral lentiginous melanoma (ALM), nodular melanoma (NM), lentigo maligna melanoma (LMM) and superficial spreading melanoma (SSM). Melanoma in early phases (mainly the radial growth phase, RGP) is entirely curable thanks to tissue excision, but if metastasis occurs the chances for patients’ survival drastically drops. The spreading of melanoma cells via the lymph nodes and vessels makes it very difficult to eliminate all tumor cells from the organism. Due to rising incidence and mortality rates, it is considered one of the top ten deadliest tumors (Forman et al. 2008; Rastrelli et al. 2014; Dummer et al. 2016; Elder 2016; Dehdashtian et al. 2018; Sobiepanek et al. 2020a).

During the multiple steps of tumor development, cells gain several biological capabilities (the so-called hallmarks of cancer), which distinguish them from normal cells. Basic hallmarks of tumor cells indicate that they are able to produce various growth factors, evade growth suppressors, gain replicative immortality, resist apoptosis, avoid immune surveillance, induce angiogenesis, and present invasive growth and formation of metastasis. Moreover, changes in the energy metabolism as well as other signaling pathways are vital parts of cell transformation and tumor formation (Hanahan and Weinberg 2011). Several metabolic pathways have been associated with melanoma metastasis including: (1) alanine, aspartate and glutamate metabolism, (2) glycine, serine and threonine metabolism, (3) arginine and proline metabolism, (4) β-alanine metabolism, (5) aminoacyl-tRNA biosynthesis, (6) cysteine and methionine metabolism and (7) d-Glutamine and d-glutamate metabolism (Kim et al. 2017). On the other hand, changes in the glycan biosynthesis pathway may lead to the altered glycosylation profile of cells which undergo tumorigenic transformation (Sweeney et al. 2018).The nature of melanoma surface glycans is highly dynamic and changes during differentiation and in response to different stimuli through the action of glycosyltransferases and glycosidases. A crucial role of some specific, modified glycans in various phases of melanoma progression has been documented (Ząbczyńska and Pocheć 2015). These changes comprise an increase in the amount of hypersialilated *N*-oligosaccharides or presence of short, simple glycans in advanced stages of melanoma (Ciołczyk-Wierzbicka et al. 2004). These properties determine glycan interactions with specific ligands, used for their identification and description of the complexes formed (Sobiepanek et al. 2017). A combined targeting of these pathways may help in establishing effective therapies for various tumors. They may also function as a set of diagnostic and prognostic biomarkers.

## General differences between melanocytes and melanoma cells

Melanocytes, which may transform into melanoma cells, originate from the neural crest, not like other skin carcinomas from embryonic epithelial cells. The key feature of the neural crest cells is multipotency and the ability to migrate. This is the main reason for the capability of melanoma cells to cross the basement membrane and metastasize (Donoghue et al. 2008; Sobiepanek et al. 2020b). Contrary to melanocytes, in which melanin synthesis is under strict control (Slominski et al. 2012), the pigment itself plays a specific biological role mainly in the skin protection against harmful factors like solar radiation, reactive species or chemotherapeutics, melanin production is highly dysregulated in melanoma cells (Mishra et al. 2014) and the amount of melanin can be a biomarker of cancer progression (Slominski et al. 2014). Degraded melanin, impaired melanogenesis or the retention of melanin in cells might have a mutagenic influence on the cells with increased melanoma appearance (Śniegocka et al. 2018).

The molecular bases of melanoma progression are the subject of intensive research. Malignant transformation is connected with mutations in genes responsible for cell proliferation and apoptosis, epigenetic changes, loss of adhesion ability or production of autocrine growth factors, which disturb the signal transduction pathways in melanocytes. For example, the first melanoma oncogene described in 1984 was the NRAS protein, which activates the mitogen-activated protein kinases MAPK) cascade (Pokrywka and Lityńska 2012). Currently, mutations in four different proteins (*BRAF, GNAQ/GNA11, cKIT, NRAS*) are well known to occur during melanoma formation, and affect the MAPK pathways by disrupting the healthy cells’ response to the emerging oncogenes. Point mutations in the v-Raf murine sarcoma viral oncogene homolog (BRAF) are present in 35–50% of melanoma cases, while in Neuroblastoma RAS viral oncogene homolog (NRAS) only in 10–25% cases, and there are loss of function mutations affecting Neurofibromin 1 (NF1) in approximately 15% of melanoma cases. Although these changes are considered as tumor markers, the unequivocal molecular marker of melanoma has not been defined yet (Scolyer et al. 2011; Fischer et al. 2018).

Metabolic changes observed in melanoma include mainly differences in the signaling pathways responsible for the cell growth, mobility, proliferation and/or the cell survival (e.g., RAS/RAF/MAPK, PI3K/AKT/mTOR and p53/Bcl-2 pathways) as compared to the normal melanocytes. On the other hand, the formation of the tumor foci in distant tissues from the primary tumor occurs through the mechanism of epithelial-mesenchymal transition (EMT). This allows the polarized epithelial type cells to modify their phenotype to mesenchymal. The cells’ adhesion ability declines, the apical-basal polarity is lost and at the same time cells gain increased mobility. The EMT process in addition to the increased mobility of cells is also accompanied by disorders in the control of the cell cycle, differentiation processes, the development of resistance to apoptosis, as well as the increased motility of cells. Angiogenesis provides a constant supply of nutrients to tumor cells, but also provides a channel through which transformed mesenchymal cells detach from the mass of the tumor and move to distant organs. Mesenchymal cells after colonizing new tissue return to the epithelial phenotype on the basis of the mesenchymal-epithelial transition (MET) mechanism (Kalluri and Weinberg 2009; Nakamura and Tokura 2011; Fenouille et al. 2012; Heerboth et al. 2015).

The significance of metabolic reprogramming in cancer initiation, maintenance, as well as progression is important. Especially when taking into consideration that this tumor is metabolically heterogeneous and capable of adapting to multiple conditions as well as to the use of a variety of fuels. There is also growing evidence that the metabolic phenotypes of melanoma cells strongly depend on not only the intrinsic oncogenic pathways but also on extrinsic factors present in the tumor microenvironment, a complex matrix composed of various cells (Hanahan and Weinberg 2011; Fischer et al. 2018).

### Metabolic pathways in melanoma

It is obvious that the central carbon metabolism is the most important process occurring in all types of cells. It consists of glycolysis, the pentose phosphate pathway (PPP) and tricarboxylic acid (TCA) cycle. Depending on the cell type (normal differentiated cells or cancer cells) glucose metabolism varies significantly. Normal cells under normoxic conditions uptake glucose, which then undergoes the process of glycolysis in the cytosol to produce pyruvate and adenosine triphosphate (ATP) molecules. Ten intracellular enzymatic reactions are required to degrade glucose into pyruvate, then the decarboxylation of pyruvate to acetyl coenzyme A (acetyl-CoA) by mitochondrial pyruvate dehydrogenase (PDH) occurs and acetyl-CoA enters the tricarboxylic acid cycle (TCA cycle, also called Krebs cycle) in mitochondria (Fischer et al. 2018). The TCA cycle produces nicotinamide adenine dinucleotide (NADH) and reduced flavin adenine dinucleotide (FADH_2_), which fuels oxidative phosphorylation (OXPHOS) by the electron transport chain (ETC) found in the mitochondrial inner wall to form ATP (Courtnay et al. 2015). Five complexes are responsible for the OXPHOS in mitochondria: ubiquinone oxidoreductase (complex I), succinate dehydrogenase (complex II), ubiquinol–cytochrome c oxidoreductase (complex III, or cytochrome bc1 complex), cytochrome c oxidase (complex IV) and ATP synthase (complex V) (Sharma et al. 2009). On the other hand, under hypoxic conditions cells rely more on glycolysis and lactic fermentation to fulfill their energy needs. This requires the transformation of glucose to pyruvate, which is then reduced to lactate by the enzyme lactate dehydrogenase (LDH), using the reducing equivalents carried by NADH (Fischer et al. 2018). OXPHOS is significantly more efficient at generating ATP than glycolysis. The total net gain of energy from a single molecule of glucose is 2 molecules of ATP gained via glycolysis or 36 ATP molecules by means of glycolysis/OXPHOS (Courtnay et al. 2015).

Nevertheless, tumor cells (including melanoma) preferentially metabolize at high rates glucose into lactate regardless of the oxygen availability, but at the same time, they are able to gather sufficient materials required for cell proliferation. This phenomenon is known as aerobic glycolysis or the “Warburg effect” (Fischer et al. 2018). Under normoxic conditions, about 25% of pyruvate enters the mitochondria of melanoma cells, whereas during hypoxic conditions this value is limited to only 7% (Scott et al. 2011). However, the mitochondria of melanoma cells stay functional and may additionally support the growth and progression of the tumor (Ruocco et al. 2019; Avagliano et al. 2020). Melanoma cells with KRAS or BRAF mutations have increased glucose uptake as well as higher glycolysis, but they are also able to survive in low glucose conditions in comparison with normal cells (Haq et al. 2013; Courtnay et al. 2015). The cell-surface GLUT-1, a member of the GLUT family of the intracellular glucose uptake membrane transport proteins, is upregulated during the reprogrammed metabolism of many tumors including melanoma and can be associated with a high tumor grade (Ruocco et al. 2019). Apart from the increased glucose consumption, metabolic rewiring in melanoma enhances the reliance on glutamine utilization (Ratnikov et al. 2017).

The activation of intrinsic signaling pathways (like MAPK or PI3K/Akt/mTOR) drives the utilization of the Warburg phenotype in melanoma cells. The activation of these signaling pathways increases the expression of the hypoxia-inducible factor 1α (*HIF1α*), v-MYC avian myelocytomatosis viral oncogene homolog (*MYC*) and microphthalmia-associated transcription factor (*MITF*) (Kumar et al. 2007; Parmenter et al. 2014; Ratnikov et al. 2017). HIF1α is the central regulator of glycolysis, cancer metabolism and cancer cell proliferation. It has a significant influence on several gene products associated with metabolism, such as: glucose transporter 1 (*GLUT1*), glucose transporter 3 (*GLUT3*), glyceraldehyde-3-P-dehydrogenase (*GAPDH*), hexokinase 1 (*HK1*), hexokinase 2 (*HK2*), lactic dehydrogenase A (*LDHA*), pyruvate kinase M (*PKM*), pyruvate dehydrogenase (*PDH*), pyruvate dehydrogenase kinase 1 (*PDC1*), carbonic anhydrase (*CA*), monocarboxylate transporter 4 (*MCT4*). For example, HIF1α activates PDK, which inhibits PDH and in this way prevents the pyruvate from entering the TCA cycle for use in OXPHOS, thus increasing lactate production (Kim et al. 2006; Stubbs and Griffiths 2010; Courtnay et al. 2015). In particular, the glycolysis induced by HIF1α is strictly linked to GLUT-1 overproduction and its localization in the membrane. The higher level of the protein GLUT-1 in cutaneous melanoma in comparison with melanocytic nevi, is positively correlated to mitotic activity, melanoma progression and metastasis (Ruocco et al. 2019). Glucose uptake may also be stimulated by MYC, which transcriptionally activates LDH, GLUT-1 and HK2 (Avagliano et al. 2020).

In various tumors, a significant amount of the glycolytic carbon is redirected into the synthesis of the non-essential amino acid serine. The Serine Synthesis Pathway (SSP) includes various enzymes beginning with phosphoglycerate dehydrogenase (PHGDH), followed by phosphoserine aminotransferase 1 (PSAT1) and phosphoserine phosphatase (PSPH). Serine, both imported from the tumor microenvironment (TME), as well as endogenously generated by the SSP, is then fueled into the one-carbon metabolism (OCM), through the activity of the key enzyme—serine hydroxymethyltransferase (SHMT). SHMT plays a relevant role in the metabolic reprogramming of cancer cells due to its overexpression. Two *SHMT* genes were identified in humans: *SHMT1* encoding the cytoplasmic isozyme (SHMT1) and *SHMT2* encoding the mitochondrial isozyme (SHMT2). Serine anabolism through OCM fuels the de novo biosynthesis of purines and pyrimidines as well as the production of antioxidant molecules like glutathione (GSH) (Amelio et al. 2014; Paone et al. 2014; Marani et al. 2016; Paiardini et al. 2016).

The control of oxidative stress plays a pivotal role in the growth of cancer cells. The Warburg phenotype is predominantly driven by a transcriptional co-factor that regulates multiple mitochondrial genes, peroxisome proliferator-activated receptor γ 1-α (PPARGC1A, also known as PGC-1α). Melanoma cells presenting a higher expression of PGC-1α are able to tolerate oxidative stress to a significantly greater extent than cells with a low expression of this factor (Vazquez et al. 2013). That is because PGC-1α can regulate reactive oxygen species (ROS) levels by inducing the expression of several enzymes involved in their detoxification, mainly superoxide dismutase 2 (SOD2) and glutathione peroxidase 1 (GPX1) (Austin and St-Pierre 2012; Luo et al. 2016). The decrease sensitivity to ROS, produced mainly during OXPHOS, is beneficial for melanoma cells, due to the fact that tumor cells during metastasis experience intense oxidative stress (Fischer et al. 2018; Avagliano et al. 2020). The nuclear peroxisome proliferator-activated receptors (PPARs) participate in the regulation of cellular metabolism, inflammation and in melanogenesis. They are a focal regulatory point at the intersection of AMP-dependent protein kinase (AMPK), mammalian target of rapamycin (mTOR) and PGC-1α signaling pathways (Grabacka et al. 2020). PPARγ activates the transcription of multiple genes involved in lipid synthesis and accumulation, and likewise it influences glucose uptake. PPARα is responsible for the transactivation genes of the peroxisomal and mitochondrial fatty acid oxidation as well as ketogenesis. The cooperation between PPARγ and α-melanocyte-stimulating hormone (α-MSH) signaling results in enhanced melanogenesis in both melanocytes and melanoma cells (Grabacka et al. 2017). PPARs are also important transcription factors taking part in the regulation of ketogenesis, which requires efficient mitochondrial β-oxidation of fatty acids. The formation of ketone bodies [including β-hydroxybutyrate (bHB), acetoacetate and acetone] supply additional substrates for the energy metabolism for certain cells from peripheral tissues apart from glucose and glutamine metabolism. Typically, melanoma cells utilize large amounts of glucose, however, it is under intensive investigation whether this monosaccharide could be replaced by ketone bodies (Grabacka et al. 2016a, b).

Differences in cell metabolism are also associated with melanogenesis. This pathway requires three melanocytic-specific enzymes: a melanosomal membrane-bound glycoprotein called tyrosinase and the tyrosinase-related proteins (TRP1 and TRP2); which are involved in the conversion of tyrosine to melanin (Ortonne and Ballotti 2000; García‐Borrón and Solano 2002). The oxidoreduction reactions also produce several intermediates including toxic compounds like quinones, semiquinones and ROS that influence the behavior of normal and malignant melanocytes and their surrounding microenvironment (Slominski et al. 2014). Melanogenesis is mediated by several melanogenic signaling pathways like p38 MAPK signaling, the cyclic adenosine monophosphate- (cAMP-) mediated pathway, the protein kinase C- (PKC-) mediated pathway, PI3K/AKT signaling, and the p44/42 MAPK pathway. That is why while affecting melanization it can directly interfere with pro-proliferation and antiapoptotic regulation; thus, melanogenesis is directly connected with the prognosis of melanoma development (Hwang et al. 2019). Furthermore, it has been found that the induction of melanogenesis in melanoma cells is associated with a dramatic increase in nuclear HIF1α expression accompanied by the upregulation of HIF1α-dependent genes involved in the regulation of glucose metabolism, angiogenesis and stress responses (Slominski et al. 2014).

Signals produced by the TME can also influence melanoma plasticity through changes in the epigenetic state to force dynamic differentiation and de-differentiation. The microenvironment-mediated epigenetic regulation of gene expression includes stress signals like pH, hypoxia, radiation, stiffness, as well as interfacial stress, which constrain a conversion of melanoma cell to a stem cell-like phenotype (Lee et al. 2020). The hypothesis of the occurrence of MTSC (melanoma tumor stem cells) presenting the CD 271 (neurotrophin receptor) antigen, responsible for metastasis occurring, has been thoroughly verified (Boiko et al. 2010). On the other hand, an oncogenic transformation may force the adaptation of cancer cells to the conditions occurring in the TME. This is done by a metabolic phenotype switch that can significantly influence the tumor microenvironment. Due to the complex TME structure, inefficient nutrient supply and oxygen delivery inside of the tumor takes place as well as poor waste removal from the tumor (Ratnikov et al. 2017; Avagliano et al. 2020; Bader et al. 2020). The growth and progression of solid tumors, like cutaneous melanoma, as well as therapeutic resistance are strongly dependent on the metabolic crosstalk between cancer cells and the TME (Ruocco et al. 2019). Lactate secretion not only drastically alters the TME, but also facilitates angiogenesis, promotes metastasis and suppresses the immune system (Romero-Garcia et al. 2016). For many years, the production of lactic acid was believed to acidify the cytoplasm of cells, affecting the tumor intracellular pH (pHi) as well as extracellular pH (pHe). In case of tumor extracellular pH, this was confirmed by the discovery of enzymes located on the cell surface membrane, which catalyze the reversible interconversion of CO_2_ to HCO_3_^−^ and H^+^, e.g., the carbonic anhydrase IX (CAIX). Carbonic anhydrases are cancer-associated cell surface zinc metalloprotein enzymes (a family of 16 isoforms), which play the key role in maintaining low tumor pHe. The pHe in healthy tissues is between 7.35 and 7.45 and it is tightly regulated to sustain the normal physiology and cellular metabolism. In case of tumor cells, pHe is acidic (6.3–7.0), reflecting the dysregulation of the acid–base homeostatic mechanisms present in solid tumors. However, it turned out that the intracellular pHi is usually neutral or alkaline in both normal and cancerous cells, which is why only pHe and its controlling factors are assumed to be potential therapeutical targets in oncology. CAIX consists of an extracellularly facing catalytic domain, an N-terminal proteoglycan-like (PG) domain, a single transmembrane domain and a short intracellular C-terminal tail, where the N-terminal PG domain of CAIX is unique within the CA family and is important in the assembly of focal adhesion contacts during cell migration (Lee and Griffiths 2020).

In addition, adherent cell types express various glycosylated cell receptors responsible for focal adhesion. Disturbance in glucose availability affects glycosylation, which is a hallmark of more than 50% of the human proteome responsible for the development, growth and survival of cells and organisms (Christiansen et al. 2014; Pinho and Reis 2015). Many studies reveal that cancer development and progression including EMT and invasion is accompanied by changes in the glycosylation patterns of cell surface and secreted glycoproteins (Munkley and Elliott 2016). Moreover, a growing amount of data has shown that different levels of oxygen tension lead to a markedly altered glycosylation, resulting in altered glycan-receptor interactions (Silva-filho et al. 2017).

### Glycosylation basics and melanoma

Glycosylation is the most common post-translational modification in cells, present in more than half of proteins synthesized (forming glycoproteins and proteoglycans) and a subset of lipids (glycosphingolipids and glycosylphosphatidylinositol anchors) (Vasconcelos-dos-Santos et al. 2015). To successfully generate these carbohydrate-associated modifications a coordinated effort of a complex array of enzymes, organelles and other factors is needed, or else the genetic and non-genetic alterations may facilitate the development of neoplastic transformation (Stowell et al. 2015). For over 35 years, the abnormal glycosylation of peptides, proteins and membrane lipids has been observed in various diseases such as diabetes, rheumatoid arthritis, multiple sclerosis and also all types of human cancers. Despite comprehensive research into the role of cancer glycobiology, including the structure and function of carbohydrates (Ghazarian et al. 2011; Dall’Olio 2012; Nardy et al. 2016), the specific characteristics of metastatic cell glycans of different types of tumors remain the subject of many studies (Häuselmann and Borsig 2014; Taniguchi and Kizuka 2015). Particular attention in such glycosylation studies is focused not only on the rapid growth and high malignancy of tumors including melanoma, but also on the identification of biomarkers or design of anti-cancer drugs.

Proteins undergo glycosylation as either a co-translational or post-translational modification (Christiansen et al. 2014). The glycosylation process involves attaching sugar groups in the form of monosaccharides or oligosaccharides to specific amino acids of the polypeptide chain (Stowell et al. 2015). So far, about 250 enzymes have been discovered that enable the glycosylation process; due to their functions, they can be divided into two groups: glycosyltransferases and glycosidases. The first group is responsible for the attachment of new sugar residues, while the second group hydrolyzes glycosidic bonds and detaches monosaccharides to trim and refine glycan side chains (Ząbczyńska and Pocheć 2015).

The majority of glycans are comprised of ten monosaccharide building blocks, which are glucose (Glc), galactose (Gal), fucose (Fuc), mannose (Man), xylose (Xyl), *N*-acetylglucosamine (GlcNAc), *N*-acetylgalactosamine (GalNAc), glucuronic acid (GlcA), iduronic acid (IdoA) and 5-*N*-acetylneuraminic acid (Neu5Ac or sialic acid) (Stowell et al. 2015). The five classes of glycans which can be created are: *N*-glycans, *O*-glycans, glycosaminoglycans (GAGs), glycosphingolipids (GSLs), and glycosylphosphatidylinositol (GPI) anchor (Marsico et al. 2018). The carbohydrate chain can be covalently bound to the protein through an oxygen atom in case of the O-glycosylation [the GalNAc residue is attached to the hydroxyl group of serine (Ser) or threonine (Thr)] or via a nitrogen atom for the N-glycosylation (the GlcNAc residue is attached to asparagine (Asn) in the amino acid sequence Asn-X-Ser/Thr, where X can be any amino acid except proline) (Christiansen et al. 2014). In case of glycosphingolipids, they are initiated by glucose addition to ceramide on the outer membrane of the ER and Golgi apparatus, and then the glycan is flipped inside these organelles to be extended. In general, the thick layer of all kinds of glycoconjugates present on the cell surface form the glycocalyx, however, some glycans secreted to the extracellular medium can also be incorporated into the extracellular matrix (ECM) (Vasconcelos-dos-Santos et al. 2015).

The donor substrates for the glycosylation process are derived in some part from the intracellular degradation of glycoconjugates in lysosomes, but mainly from extracellular glucose. During the glycolysis process, a side path may be activated, in which the glucose metabolite can be diverted to the Hexosamine Biosynthetic Pathway (HBP). Normal cells direct 2–5% of glucose to the HBP pathway, while for tumor cells this amount is altered mainly by the fact that cancer cells utilize far more glucose than normal cells (Akella et al. 2019). Fructose-6-phosphate (Fru-6P) can be converted to glucosamine-6-phosphate (GlcN-6P) by glutamine fructose-6-phosphate aminotransferase (GFAT) using glutamine (Gln) as an amine donor. Next, GlcN-6P is further metabolized to uridine-5′-diphosphate-*N*-acetylglucosamine (UDP-GlcNAc), which serves as a major substrate for several kinds of glycosylations (Vasconcelos-dos-Santos et al. 2015). The glycan biosynthesis carries on with the addition of UDP-GlcNAc or its derivatives to dolichol phosphate (in case of *N*-/*O*-glycans) or ceramide (Cer; in case of glycosphingolipids) (Stanley et al. 2017; Schnaar and Kinoshita 2017). The process of *N*- and *O*-glycan oligosaccharide synthesis takes place on the membrane of the endoplasmic reticulum (ER). After several enzyme activities, the glycan structure bonds to the folded protein and is directed to the Golgi apparatus. There, the *N*-glycan takes its final form of a pentasaccharide core with high mannose chains, and hybrid or complex type side chains. In case of the *O*-glycans, they are built from different types of cores (*O*-GalNAc, *O*-Man, o-Fuc and o-Glc) with a few side chains (Vasconcelos-dos-Santos et al. 2015).

The altered glycosylation of cell-surface proteins, which is observed at all stages of cancer progression, results in the increased growth, proliferation and survival of cells as well as gives cancer cells the ability to migrate and invade. The accumulation of genetic and epigenetic changes results in dysregulated transcription, which causes an altered expression and also altered activity or the mislocalization of enzymes engaged in the glycan biosynthesis pathway. Typical cancer-associated changes in glycosylation include: (1) the overexpression and underexpression of naturally occurring glycans, (2) the expression of glycans normally restricted to embryonic tissues, (3) the appearance of incomplete or truncated structures and also (4) the appearance of novel structures (less frequently) (Link-Lenczowski and Lityńska 2011; Hoja-Łukowicz et al. 2017). Although it is well known that aberrant glycans are present on various cancer cells, the regulation of glycosylation patterns in different cancers is still largely unknown (Sweeney et al. 2018). In case of melanoma, the most common modification is a higher expression of β1,6-branched N-linked glycans (responsible for a significant enhancement of cell motility through the ECM substrates) caused by the increased transcription and activity of β1,6-N-acetylglucosaminyltransferase V (GnT-V). Another upregulated enzyme responsible for the increased expression of bisected *N*-glycans is β1,4-*N*-acetylglucosaminyltransferase III (GnT-III), which may promote cancer progression. Likewise, the enhanced activity of sialyltransferases such as α2–3 and α2–6 was observed in melanoma cells. Accelerated cell-surface sialylation and/or differences in the position of sialic residues, including α2,8-linked polysialic acid (PolySia), are responsible for the modulation of cell recognition, cell adhesion and cell–cell signaling. The accumulation of the negative charge in the form of sialic acid content on the surface of the cell membrane causes repulsion between cells, reduces tumor cell anchorage to ECM components, and finally allows tumor cells to migrate from the tumor site. The truncation of serine/threonine *O*-linked glycans with T (Galβ1–3GalNAc-α1-*O*-Ser/Thr), Tn (GalNAc-α1-*O*-Ser/Thr), and sialyl-Tn (Siaα2-6GalNAc-α1-*O*-Ser/Thr; sTn) mucin antigens was only observed in tumor tissues. Thus, it may be a good diagnostic marker, but not a prognostic marker due to the presence of *O*-glycan changes occurring independently from the cancer progression stage (Lityńska et al 2001; Ciołczyk-Wierzbicka et al. 2004; Przybyło et al. 2007; Pinho and Reis 2015; Lucena et al. 2016; Hoja-Łukowicz et al. 2017).

Melanoma cells also show an increase in the expression of the cell-adhesion molecules (CAMs), which are receptors located on the cell surface and involved in the binding of cell-to-cell and cell-to-ECM. The major difference in their expression between normal and tumor cells was observed in case of proteins like L1 cell-adhesion molecule (L1-CAM), melanoma CAM (MCAM), activated leucocyte CAM (ALCAM), vascular CAM-1 (VCAM-1), intercellular adhesion molecule 1 (ICAM-1), and integrins as well as cadherins. Integrins are highly glycosylated proteins responsible for ligand binding to for example fibronectin and collagen, complexation with other membrane proteins and signal transduction by recruiting the focal adhesion kinase (FAK) or Src family kinases. Thus, integrins can initiate pro-survival but also pro-apoptotic signals. They play an important role at every stage of cancer development, where disturbance in their adhesion is caused by aberrant N-glycosylation. Compared to normal melanocytes, melanoma cells show overexpression of α1β1, α2β1, α3β1, α4β1, α5β1, α6β4, αvβ3 αvβ6 and a loss of α6β1 integrins. It is worth emphasizing that some of these changes are characteristics of the metastasis process, e.g., integrin αvβ3 is considered a marker of the RGP-to-VGP transition. In contrast, α1β1 and α2β1 integrins occur only on the surface of metastatic melanoma cells but α3β1 is present on both types of melanoma cells: primary and metastatic (Link-Lenczowski and Lityńska 2011; Läubli and Borsig 2019). The cadherins are a different type of cell surface protein and play a significant role in epithelial intercellular adhesion. Disturbances in cadherin adhesion may lead to the formation of a tumor originating from epithelial cells: normal melanocytes express E-cadherin and P-cadherin, where E-cadherin ensures the adhesion of melanocytes to keratinocytes (Przybyło et al. 2002; Ciołczyk-Wierzbicka et al. 2004). This contact enables melanin transfer from melanocytes to keratinocytes through three different mechanisms (Ando et al. 2012), one of which involves the formation of a structure called a pigmentation synapse (the melanocyte–keratinocyte adhesion site), where the engagement of surface glycans and lectins is a factor responsible for melanin transfer (Van Den Bossche et al. 2006). During oncogenesis, keratinocyte control over melanocytes is reduced and N-cadherin seems to replace the normal E-cadherin especially in tumor cells with a high level of invasiveness. This process is known as the cadherin-switch. The increasing amount of N-cadherin with a simultaneous loss of the ability to produce E-cadherin is a factor that causes a break in the binding between melanocytes and keratinocytes. The effect of this phenomenon is the acquisition of new adhesion properties by these cells. N-cadherin significantly affects the strength of intercellular and intracellular interactions. However, N-cadherin not only initiates the binding between cancer cells of melanocytic origin and fibroblasts or epithelial cells, but also supports the survival and migration of cancer cells. The switch in cadherins is considered to be the most likely cause of tumor formation (Przybyło et al. 2002; Ciołczyk-Wierzbicka et al. 2004; Sobiepanek et al. 2020a). This phenomenon could also be associated with the production of glycosylation structures known as β1,6-branched oligosaccharides. These structures are not produced by normal melanocytes or by cells of early melanoma in situ, but characterize invasive and metastatic tumors. The aberrant expression of β1,6-branched oligosaccharides is associated with the increased melanin production, autophagy and high invasive potential of melanoma cells, while in normal melanocytes the accumulation of melanin would cause apoptosis (Lazova and Pawelek 2009).

## Methodology used for investigating changes in melanoma signaling pathways

**Fig. 1:**
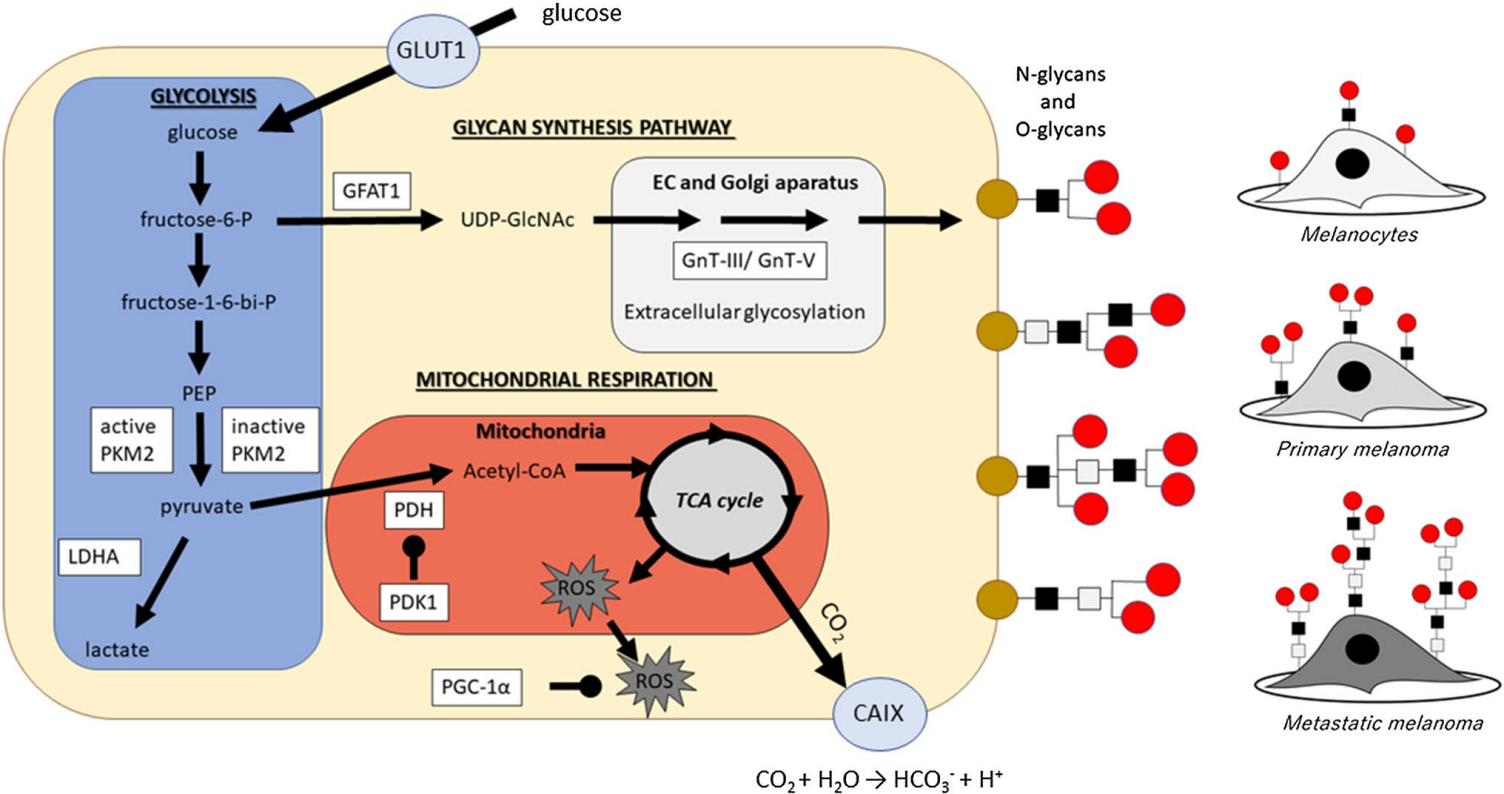
The connection between various pathways in cells: glycolysis, OXPHOS, TME signaling, HBP and glycan biosynthesis pathway

Taking into account that most metabolism and signaling pathways are connected (Fig. 1), the reprogramming of one pathway may have a crucial impact on others. Therefore, the knowledge of methods allowing a detailed analysis of each pathway can help in establishing an effective treatment of several diseases including melanoma skin cancer.

### Analysis of energy metabolism in melanoma cells

Glucose and glutamine uptake regulates the metabolism of a whole cell and for various tumors has been found to be significantly increased. A dynamic picture of metabolism may be explored thanks to stable isotope (^13^C or ^14^C) tracing (Allen and Young 2020). The growth medium can be supplemented with the selected concentration of [U-^13^C6] glucose or/and [U-^13^C5] glutamine before adding it to the cells, to give labeling that should last for at least a couple of hours. For metastatic melanoma cell line A375, the described physiologically relevant concentration of [1,2-^13^C] glucose was 5 mM (Delgado-Goñi et al. 2020). Medium samples should be collected at the start (0 h) and after cell treatment (e.g., 24 or 48 h). Then, the cell should be collected via trypsinization, rinsed with a PBS buffer and centrifuged to obtain cell pellets, which should be stored at − 80 °C. Sample preparation for gas chromatography combined with mass spectrometry (GC–MS) (Scott et al. 2011) or for nuclear magnetic resonance (NMR) spectroscopy (Wang et al. 2014; Delgado-Goñi et al. 2020) analyses is performed by the methanol-chloroform extraction of the analytes like glucose, pyruvate or fatty acids from the media samples or cell pellets. A simpler analysis can be also performed with the use of commercial kits for the quantification of glucose and glutamine consumption as well as lactate production. These assays are also based on medium collection from samples and next by the addition of the appropriate enzyme (e.g., hexokinase, glucose 6-phosphate dehydrogenase, glutamate dehydrogenase, lactate dehydrogenase) provided by the manufacturer to the sample. After 30 min incubation time, an absorbance or fluorescence measurement is taken (Scott et al. 2011; Gkiouli et al. 2019). With such approaches, for example, a comparative profiling of metabolic fluxes in melanocytes (H3A and NEM-LP) and melanoma cell lines (WM35, Mel501, UACC903, WM793, Lu1205 and MeWo) grown under normoxic and hypoxic conditions was performed. Melanoma cells presented mainly the Warburg effect, but not all glucose was consumed during glycolysis. Glutamine was especially important for fatty acid synthesis under hypoxia (Scott et al. 2011). Furthermore, the GC–MS analysis allowed identification of up to 39 metabolites (including alcohols, amino acids organic acids, purines, pyrimidines, sugars and other metabolites) of human epidermal melanocytes (HEMn-LP) and two melanoma cell lines (A375, A2058) with a different stage of metastasis (Kim et al. 2017). The ^1^HNMR-based metabolomics analysis of metastatic melanoma in C57BL/6J mouse spleen showed that the selected metabolites occurrence in samples with tumor cells can be well separated from that observed with normal cells. Melanoma samples presented decreased amounts of taurine, glutamate, aspartate, *O*-phosphoethanolamine, niacinamide, ATP, lipids and glycerol derivatives; as well as increased amounts of alanine, malate, xanthine, histamine, deoxycytidine triphosphate, guanosine triphosphate, thymidine and 2′-deoxyguanosine (Wang et al. 2014).

Changes in cellular metabolism can also be observed following the gene expression and enzyme activity by qPCR or Western blot/ELISA techniques, respectively. From the cancer research point of view, some of the promising metabolic targets are proteins connected to glycolysis (GLUT1, GAPDH, PKM2, LDHA, MCT1), glutamine metabolism (xCT, GLS), fatty acids synthesis (CPT1, MGLL), pyruvate metabolism (PDK1, PDC) and other regulators (HIF, mTOR, CAIX) (Kim et al. 2006; Stubbs and Griffiths 2010; Courtnay et al. 2015; Ratnikov et al. 2017). For these analyses, cells are collected by trypsinization and RNA is obtained from cells by means of guanidinium thiocyanate–phenol–chloroform extraction (Chomzynski 1987). After quantitative determination of the RNA concentration (the absorbance read at 260/280 nm and 260/230 nm) and its qualitative analysis (horizontal electrophoresis in an agarose gel), reverse transcription is performed and real-time polymerase chain reactions (qPCR) performed with primers corresponding to the investigated molecular targets. In case of Western blot or ELISA techniques, the proteins are isolated from cells collected by trypsinization or mechanical scraping (depends on the molecular targets of the investigation), cell centrifugation, washing with a phosphate-buffered saline (PBS) buffer and a secondary centrifugation to obtain cell pellets, which should be stored at − 20 °C. After the protein extraction from cells, their content in samples is determined usually with the BCA Protein Assay Kit or Bradford reagent on a microplate reader. Different concentrations of bovine serum albumin (BSA) are used for the preparation of the standard curve (Eslami and Lujan 2010). Then, the samples may be provided for the electrotransfer or ELISA assay, which is followed by the incubation of the received membrane (e.g., polyvinylidene fluoride—PVDF, nitrocellulose) or multi-well plate with the blocking solution, and next with the primary and secondary antibodies (conjugated with an enzyme or fluorophore). For antibodies conjugated with enzymes, the corresponding substrate must be used. The results are obtained from the visualization of bands via absorbance/fluorescence (Western blot) or each single-well read from the whole plate (ELISA) (Andreucci et al. 2018). From such studies primary and metastatic melanoma tissues have shown an enhanced expression of GLUT1 transporter, which promoted glucose uptake and cell progression, compared to the tissue with benign nevi. Furthermore, the suppression of *GLUT1* in melanoma cells with shRNA revealed significantly reduced proliferation, apoptosis resistance, migratory activity and matrix metalloproteinase 2 (MMP-2) expression (Koch et al. 2015). The qPCR/Western blot investigation, performed on patient-derived melanoma cells, showed an increased *HIF1α* expression in comparison with normal melanocytes. The presence of BRAF (V600E) mutation in melanoma cells increased the expression of *HIF1α* as well as cell survival under hypoxic conditions (Kumar et al. 2007). In another study, three BRAF-mutant melanoma cell lines (M14, A375 and 518A2) cultured under hypoxia presented not only the overexpression of *HIF1α*, but also of *CAIX* (Pucciarelli et al. 2016). Melanoma cells with BRAF (V600E) mutation also suppressed the expression of the mitochondrial metabolism regulator—PGC-1α (Haq et al. 2013). The transcription factor SOX2 (sex-determining region Y (SRY)-Box2) is correlated with growth, tumorigenicity as well as drug resistance and its increased expression was observed in melanoma cells. Moreover, SOX2 protein occurrence was significantly increased in A375-M6 melanoma cells exposed to acidic medium (pH 6.7) in comparison with the neutral medium (pH 7.4). This additionally induced a metabolic shift towards oxidative phosphorylation with a simultaneous slowdown of the glycolytic pathway, as confirmed by the reduced lactate production (Andreucci et al. 2018). Since hypoxia‑induced changes in metabolism can affect the extracellular pH and oxygen consumption of cells, these parameters should be monitored, for example, in real-time by a non‑invasive SDR optical sensor system (Pucciarelli et al. 2016).

On the other hand, the most advanced analysis of the in vitro cell metabolism may be performed by means of the Extracellular Flux Analyzer from Seahorse Bioscience. The XF Cell Mito Stress Kit allows the measurement of the efficiency of glycolysis and the mitochondrial oxidative phosphorylation. The measured parameters are the oxygen consumption rate (OCR) and the extracellular acidification rate (ECAR). The primary set up of the conditions required for the Seahorse experiments with different cell types includes: the appropriate cell number per well and the selection of drug concentrations necessary for the correct performance of the Seahorse analysis. The Seahorse drugs are: oligomycin—an ATP synthase inhibitor, carbonyl cyanide-4-(trifluoromethoxy) phenylhydrazone (FCCP)—the protonophoric uncoupler and a mixture of Rotenone with Antimicin A (Rot/AA), which functions as the electron transport inhibitor. To enable the normalisation of the cell number in each well, one of two methods should be applied (cell counting or the total protein determination) on the plates after the Seahorse experiments (Zhang and Zhang 2019). Various melanoma cell lines (A375, ED-007, ED-013, ED-024, ED-027, ED-029, ED-034, ED-050, ED-070, ED-071, ED-117, ED-140, ED-179 and ED-196, MEL103, MEL526, and MEL697, Melmet 1, Melmet 5 and SK-MEL-28) showed higher glycolytic rates (higher basal glycolytic ECARs) compared to normal melanocytes, as well as higher maximal glycolytic capacities. In general, there were no significant differences in the basal and maximal mitochondrial respiration between melanocytes and melanoma cells (OCR) (Abildgaard et al. 2014; Bettum et al. 2015; Shankar Babu et al. 2018).

The natural by-products of cell metabolism, like reactive oxygen (ROS) and nitrogen (RNS) species, play significant roles in cell signaling and homeostasis, as well as allowing measurement of changes in metabolism (Ozcan and Ogun 2015; Ciccarese and Ciminale 2017). Accumulation of ROS in cells has been recognized as one of the mechanisms leading to DNA damage associated with mitochondrial dysfunction and apoptosis (Kwon et al. 2019). To detect ROS and RNS several analytical approaches have been used, including electron paramagnetic resonance (EPR), chemiluminescence and fluorescence. For example, measurement of the intracellular and mitochondrial superoxide (O_2_^·−^) can be performed using hydroethidine (HE) and Mito-SOX. For establishing the amount of the released peroxynitrite (ONOO^−^) the most frequently used probe is dihydrorhodamine (DHR). Dichlorodihydrofluorescein diacetate (DCFH-DA) may be used for the determination of intracellular hydrogen peroxide (H_2_O_2_), but this assay has several limitations and artifacts associated with the experiment (more details in Kalyanaraman et al. 2012). The optimization of these assays is connected with the selection of the proper number of cells per well and also the concentration of the labeling marker for each cell line individually. As positive controls extracellular H_2_O_2_ or *tert*-butyl hydroperoxide (*t*-BHP) may be used in a cell-dependent manner, and to reduce the amount of reactive species production GSH may be applied (Slamenova et al. 2013; Kwon et al. 2019). The capability of suppressing increased ROS levels was confirmed only in melanocytes. Therefore, the increased ROS level occurrence in melanoma cells has been confirmed so far through multiple mechanisms (de Melo et al. 2013; Liu-Smith et al. 2014).

An important issue is also the metabolic plasticity of cancer cells (Avagliano et al. 2020; Rodríguez-Enríquez et al. 2020). Atomic force microscopy (AFM) in the force spectroscopy mode is widely used for measuring mechanical properties of cells in their physiological conditions in vitro. From the AFM measurements, force-distance curves are analyzed and the Young’s modulus (in some cases called elastic modulus) is typically calculated using the Hertz–Sneddon model, which includes the geometry of the tip (Lekka 2017). Mechanical properties of cells mainly correspond to the organization of the cell cytoskeleton, especially actin filaments at the indentation depth up to 500 nm (Pogoda et al. 2012; Gostek et al. 2015). In case of melanoma cells, various research has shown significant differences between normal cells and malignant cells, where normal cells obtain higher values of cells’ elastic values (Lekka et al. 1999, 2012). This is also the issue for melanocytes and melanoma cells (Sobiepanek et al. 2017); however, cell stiffness is a dynamic property of the primary and metastatic melanoma cell lines, thus the ability to vary their degree of elasticity towards both very low and very high values is a marker of malignancy (Weder et al. 2014). Therefore, changes in melanoma cell stiffness cannot be interpreted in a simple way and the use of AFM analysis for diagnostic purposes in case of patients with melanoma should be approached with extreme caution. Yet, some attempts have also been made to relate the dependence of melanin load of cells with the mechanical properties of melanoma cells (Sarna et al. 2018). The presence of melanin granules dramatically modified the elastic properties of the pigmented melanoma cells, where the non-pigmented cells presented the lowest values of Young’s modulus (ca. 2 kPa) and heavily pigmented the highest values (ca. 7 kPa) (Sarna et al. 2014). On the other hand, melanoma cells containing melanin were less capable of spread in mice than cells without the pigment, which indicates that the presence of melanin inhibits melanoma metastasis (Sarna et al. 2019). The melanin content in cells could also be determined by electron paramagnetic resonance (EPR) spectroscopy based on the intensity and spectral parameters of characteristic EPR signals of eumelanin and pheomelanin (Sarna et al. 2014). Using a single scan with EPR the effect of free radicals in melanin and melanoma cells can be obtained (Pilawa et al. 2017; Żądło 2019). The ability of EPR imaging to accurately map melanoma depends on the concentration of melanin in the sample, which is proportional to the growth stage of the tumor (Godechal et al. 2012).

Finally, an indirect analysis to study changes in cell metabolism is conducted by means of cell migration tests. For this purpose, a transwell migration/invasion assay, wound-healing assay or individual cell-tracking systems may be utilized. Although the inserts for the transwell migration/invasion assays are quite expensive, the test brings further knowledge of cell behavior than the simple wound-healing assay. Concerning the creation of the wound in the cell culture, it can be done manually using a pipette tip (in this case it is called the scratch assay) or first a silicon insert may be placed in the culture dish and then the suspension of cells is added. Contrary to other tests, the individual cell-tracking requires cell seeding at low density. Furthermore, the analysis requires the use of a special type of microscope equipped with a cage incubator (at 37 °C and 5% CO_2_) and performing time-lapse imaging to collect a large amount of data for analysis. Melanoma cells feature a highly migratory phenotype facilitating the colonization of distant organs like lung, liver, spleen or brain (Zhu et al. 2004; Pucciarelli et al. 2016; Pijuan et al. 2019). On the other hand, cell migration is also strongly influenced by the glycosylation of cells.

### Analysis of melanoma cell glycosylation

Changes in the glycosylation pattern of surface glycans lead to the preferential occurrence of certain types of glycoconjugates in several diseases including cancer. Within several years many glycosylation-related disorders have been identified and assigned to a proper class of glycans (N-linked, glycosaminoglycan, dystroglycanopathy, *O*-GalNAc/*O*-GlcNAc, GPI-anchor, glycolipid, *O*-fucose/*O*-glucose) (Freeze et al. 2014). These differences appear mainly due to changes in glycosyltransferases and glycosidases, which regulate the glycan homeostasis (Taniguchi and Kizuka 2015). Similarities and differences in the carbohydrate chains of *N*- and *O*-glycans present on the cell surface are specific for particular phases of cancer development. Depending on the glycosylation changes, the following processes can have different advances: adhesion to endothelium, cell–cell adhesion, cell motility, immune system modulation, growth factor modulation and growth receptor regulation (Potapenko et al. 2010). Thus, one of the options for investigating the possible changes occurring in the glycosylation of cells is by means of the expression of genes encoding enzymes responsible for the glycan biosynthesis pathway or by checking enzyme activity. However, because glycan biosynthesis is a complex process, which consists of several steps to receive the final form of *N*-glycans or *O*-glycans with side chains, the selection of the gene/enzyme should be made with caution (Vasconcelos-dos-Santos et al. 2015). Some of the crucial enzymes are glutamine fructose-6-phosphate aminotransferase (GFAT) and dolichyl-phosphate mannosyl transferase (also known as dolichol-phosphate mannose synthase, DPMs). Glutamine fructose-6-phosphate aminotransferase-1 (GFAT-1) is one of the two main isoforms of the GFAT family, which converts fructose 6-phosphate to glucosamine 6-phosphate and it is the rate-limiting enzyme of the HBP pathway (Gélinas et al. 2018). Dolichyl-Phosphate Mannosyl transferase Subunit 1 (DPM1) is the largest subunit of the DPMs, which represents the catalytic center of the enzyme that generates dolichol phosphate-mannose (Dol-P-Man) from GDP-mannose (GDP-Man) and dolichol phosphate (Dol-P) (Aebi and Hennet 2001). These two enzymes are essential for the first few steps of glycan biosynthesis; thus, a differential expression of genes encoding them may show differences in the degree of cell glycosylation as well as a possible switch of glucose metabolism to the HBP pathway. Furthermore, the skin cutaneous melanoma has shown the upregulation of the HBP pathway activity as well as single HBP components in comparison with the normal skin (Jia et al. 2020). On the other hand, the most important enzymes responsible for the *N*-glycan branching are GnT-III (encoded by gene *MGAT3*), GnT-IV (encoded be two genes *MGAT4a* and *MGAT4b*), GnT-V (encoded by gene *MGAT5*), GnT-IX (encoded by gene *MGAT9*), Fut8 (encoded by gene *FUT8*) and GCNT2 (Taniguchi and Kizuka 2015; Harada et al. 2019; Dimitroff 2019). The overexpression of *MGAT3* and *MGAT5* genes were identified in metastatic melanoma cell line WM266-4 isolated from the lymph node (Bubka et al. 2014; Link-Lenczowski et al. 2018). The primary and metastatic melanoma specimens present a strong inverse relationship between GCNT2 expression and metastases. With increasing metastatic potential, cells mostly lacked the I-branched N-glycan antennae which was due to the depressed GCNT2 expression (Sweeney et al. 2018). In general, the results obtained for melanoma cells demonstrate that the core structure profile of N-glycans may be inherited at the genetic level during the multiple selection cycles of B16 variants (B16–F1, B16–F10, B16–BL6) (Harada et al. 2019).

On the other hand, the carbohydrate structures may be detected by lectins, proteins or glycoproteins of a non-immunological origin, commonly found in viruses, bacteria, fungi, plants, animals and humans. Their characteristic feature is the ability to bind specifically and reversibly distinct mono- and oligosaccharides without the use of enzymes. The lectin-carbohydrate binding is obtained through non-covalent interactions like hydrogen bonds, van der Waals forces and hydrophobic interactions. Binding with carbohydrates located on the cells’ surface ensures the occurrence of the connection between cells (Coelho et al. 2017). Due to the multivalent nature of these interactions, lectins have a high affinity for the spatial structures of oligosaccharides (Yakovleva et al. 2009). Unlike most glycoproteins, due to the presence of at least two binding sites, they are capable of agglutinating or precipitating glycoconjugates (Peumans and Van Damme 1995). Some of the typically used lectins are: concanavalin A (Con A; specific for α-d-mannose and α-d-glucose, *Helix pomatia* agglutinin (HPA; *N*-acetylgalactosamine), Peanut agglutinin (PNA; β-galactose), *Ricinus communis* agglutinin (RCA; β-d-galactose), Soybean agglutinin (SBA; galactose/α- and β-*N*-acetylgalactosamine), *Ulex europaeus* agglutinin-I (UEA-I; l-fucose) and Wheat germ agglutinin (WGA, *N*-acetylglucosamine) (Wu et al. 2008; Sobiepanek 2017).

Some studies using lectins to detect specific sugars have shown that the detection of altered glycoconjugates can help to distinguish abnormal cells from normal ones (Laidler et al. 2006). To study this characteristic effect, techniques like Western blotting (Przybyło et al. 2002; Hoja-Łukowicz et al. 2014), fluorescence microscopy (Peiris et al. 2012), enzyme-linked lectin sorbent assays (ELLSA) (Wu et al. 2008), structural studies by X-ray crystallography and nuclear magnetic resonance (NMR) (Henrichsen et al. 2004), high-performance liquid chromatography (HPLC) (Taniguchi and Kizuka 2015), affinity chromatography (HPLAC), affinity capillary electrophoresis (ACE) (Lebed et al. 2007), electrochemical impedance spectroscopy (EIS) (Carvalho et al. 2014) and several mass spectrometry techniques (ICP-MS, MALDI–MS, LC–MS/MS) (Ciołczyk-Wierzbicka et al. 2004; Gao et al. 2016; Zhou et al. 2017) may be used. Although highly informative, these methods are mostly destructive for the samples; likewise, they require expensive and time-consuming preparations (Sobiepanek 2017). For example, the structural analysis of glycans present on the melanoma cell surface by means of MALDI–TOF–MS is achieved by: cell harvesting, homogenization via sonication, immunoprecipitation, electrotransfer, deglycosylation, sugar extraction, esterification of sialic acid, microcolumn clean-up of sugars, recording of the matrix-assisted laser description ionisation mass spectra and finally detailed data analysis with some basic assumptions for these kinds of results (e.g., specified algorithms). This approach has provided interesting insights concerning the glycosylation pattern of N-cadherins on melanoma cells. Cells from the primary tumor site (WM35—RGP) possessed high-mannose and biantennary complex type glycans with α2–6 linked sialic acid, whereas different metastatic cell lines (WM239—skin, WM9—the lymph nodes and A375—solid tumor) possessed mostly tri- or tetra-antennary complex type glycans. Furthermore, N-cadherin in some metastatic cell lines contained heavily α-fucosylated complex type chains with α2,3 linked sialic acid (Ciołczyk-Wierzbicka et al. 2004). However, during the MS analysis some monosaccharides may be removed from the glycan structure due to the weakness of the glycosidic bonds, thus the structures inferred may not be identical with the real ones (Zhou et al 2017). *N*-Glycans may be also released from cells by PNGase F digestion, and then fluorescently labelled and separated by anion-exchange HPLC. Glycans may be analysed by reverse-phase HPLC or MS to determine their structure. The relative amounts of complex-type, hybrid-type and high-mannose-type glycans in melanoma cells B16–F10 were found to be 62%, 1% and 37%, respectively (Harada et al. 2019).

**Fig. 2:**
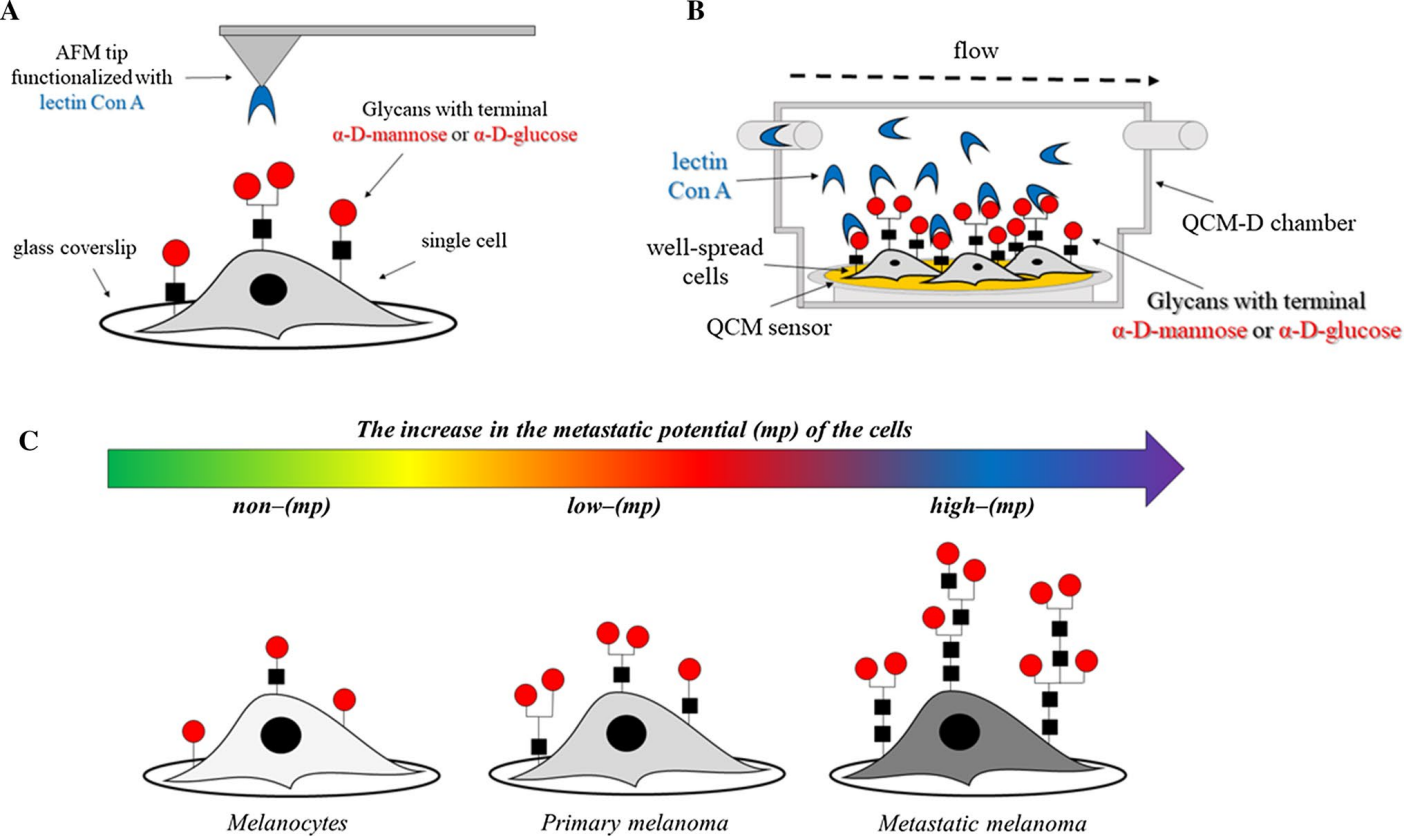
Biophysical characterization of glycans present on melanocytes/melanoma cells by means of label-free methods: AFM after tip functionalization with lectin (**a**) and QCM-D during the measurement with lectin (**b**). Schematic representation of the distinction of melanoma cells with a higher metastatic potential from the cells with a lower metastatic potential based on the performed measurements of lectin binding to glycans on cells by means of AFM/QCM-D (**c**)

Moreover, label-free nanotechnologies such as atomic force microscopy (AFM), quartz crystal microbalance (QCM) and quartz crystal microbalance with dissipation energy monitoring (QCM-D) (Lebed et al. 2007; Pei et al. 2012; Sobiepanek et al. 2017), surface plasmon resonance (SPR) or isothermal titration calorimetry (ITC) (Li et al. 2015) are also increasingly gaining attention in the study of interactions between lectins and glycans on cells. They enable not only the detection of the selected glycoconjugates, but also the determination of the kinetic and thermodynamic parameters of the interactions occurring as well as their affinity. Some methods require not only the isolation of glycans, but also purification of the selected target (for SPR and ITC); whereas others can use whole cells with glycan structures present on their membranes as targets (AFM and QCM/QCM-D). The integral membrane proteins require a lipid bilayer environment to maintain their structure and function, thus methods which do not require the isolation of glycoconjugates from cells give more reliable results imitating a biologically relevant environment for these molecules. Furthermore, to obtain the appropriate concentration of glycans in solution, the tissue dimension must be of a sufficient size otherwise the SPR and ITC measurements will not be possible (Katrlík et al. 2010; Li et al. 2015; Sobiepanek and Kobiela 2018). In AFM microscopy, the lectin must be immobilized on the AFM tip and during the measurement it interacts with the glycans present on a single cell (Fig. 2a), but during QCM/QCM-D measurements the lectin is introduced in the buffer solution from where it can directly bind with specific glycans present on all cells cultured on the surface of the sensor (Fig. 2b) (Sobiepanek et al. 2017). In general, the optimization of these measurements is concentrated on selecting: the proper buffer, type of surface for cell growth and the number of cells cultured on these surfaces (Sobiepanek and Kobiela 2021). A properly established measurement protocol allows one to obtain information about the occurring lectin-glycan interactions. For example, the glycosylation level on melanoma cell surfaces was probed using lectin concanavalin A and the QCM-D/AFM methods. The interactions of Con A with surface glycans were quantified and showed a higher affinity of the studied lectin to glycans on metastatic cells than on cells from the primary site. These measurements revealed the presence of various glycan structures in a cell-dependent manner and with different viscoelastic properties. The mannose or glucose types of glycans present on cells from the primary tumor site (WM35) were rather short and less branched while on the metastatic cells (A375-P) Con A was bound to long, branched types of oligosaccharides (Fig. 2c) (Sobiepanek et al. 2017).

## Compounds used for the reprogramming of melanoma cells

Due to the fact that melanoma is highly heterogeneous, the application of a single drug-therapy may be insufficient. Therefore, for use in a combined therapy, the knowledge of each new drug/compound mechanism of action should be first gained separately. It is extremely important to obtain satisfying results with in vitro and in vivo models before requesting approval of the Food and Drug Administration (FDA). Several groups of compounds may be mentioned in case of melanoma reprogramming treatment beginning with inhibitors of various kinases, through to effectors of metabolism, and also by using endogenous as well as exogenous molecules.

### Kinase inhibitors

Metabolic dysregulation in melanoma is modulated by oncogenic activation. The most common genetic alterations are activating mutations in the BRAF proto-oncogene. It is described that inhibition of BRAF kinase leads to a decrease in glucose uptake both in cell culture and in melanoma patients (Haq 2014; Rankin et al. 2016; Wigerup et al. 2016). Thus, the adaptive response to BRAF/MEK inhibitors (BRAFi/MEKi) is associated with the shift from glycolysis toward oxidative phosphorylation (Avagliano et al. 2020).

One of the best-known inhibitors of the BRAF kinase is Vemurafenib (VEM, PLX4032)—approved in 2011 by FDA, but the majority of patients relapse within 6–7 months of treatment. This is possibly due to the ability of melanoma cells to undergo reprogramming, leading to the survival of some tumor cells. Dabrafenib (GSK2118436) is a second best known selective BRAF-mutant inhibitor approved in 2013 (Parmenter et al. 2014; Domingues et al. 2018).

The in vitro established BRAFi-resistant clones of melanoma cells were shown to produce significantly less TCA metabolism-related metabolites (acetate, fumarate), PPP metabolites (ribose), glutamine (glutamine, glutamate, glutathione) and HBP metabolites (UDP-GlcNAC) by NMR metabolic analysis. At the same time, it was shown that BRAF-mutant melanoma cells, which were sensitive to BRAF inhibitors, presented higher glycolytic, bioenergetic and phospholipid metabolic activity compared to resistant cells. Also, the [1,2-^13^C] glucose administration revealed that BRAFi-sensitive cells had a significantly higher glucose uptake in comparison with drug resistant cells (Delgado-Goñi et al. 2020).

Gene expression and protein level/enzyme activity analysis brought a great deal of information about the influence of vemurafenib on different melanoma cells. Normally, melanoma cells with activation of the BRAF/MAPK pathway (M14, MALME-3M, UACC-62, UACC257) presented suppressed levels of *MITF* and *PGC-1α*—the master regulator of the mitochondrial biogenesis and OXPHOS. However, after cell treatment with VEM, *PGC-1α* expression was induced even up to 3–14 fold. Nevertheless, VEM had no influence on *PGC-1α* gene expression in case of wild-type melanoma cells (MeWo) treatment (Haq et al. 2013). Vemurafenib‑treated A375 cells under hypoxic conditions also exhibited an enhanced cell proliferation rate and migratory capacity compared to the normoxic vemurafenib‑treated A375 cells. The expression of *HIF1α* and *CAIX* were reduced in vemurafenib‑treated M14 and 518A2 cells, but not in A375 cells (all three cell lines are BRAF-mutants) (Pucciarelli et al. 2016). BRAF-mutant melanoma cells (A375) also presented the induced expression of *GLUT-1*, which in line enhanced glucose transport into these cells (Delgado-Goñi et al. 2020). The inhibition of the BRAF-mutant cells by the VEM treatment suppressed glycolysis independently of cell cycle progression and cell death via the suppression of the expression of HK2 as well as glucose transporter-1 and -3 (GLUT1/3) (Parmenter et al. 2014). But when establishing VEM-resistant clones of the BRAF-mutant cell lines, they not only expressed reduced levels of *GLUT-1* (Delgado-Goñi et al. 2020), but also the glucose metabolism was restored (Parmenter et al. 2014).

### Effectors of metabolism

Nowadays, a popular therapeutic strategy for cancer treatment is to apply drugs which attack different functions of mitochondrial metabolism (bioenergetics, signaling and biosynthesis). This can change the TCA activity of cells and lead to a strong energetic stress in tumor cells inhibiting other pathways (like mTOR, PPP and HBP) and inducing autophagy (Missiroli et al. 2020). But the reprogramming of tumor cell metabolism is a very complex and heterogeneous process, which is influenced by a wide variety of genetic and non-genetic strategies (Abildgaard et al. 2014). Most of the available drugs are inhibitors of the electron transport chain complexes in mitochondria (complex I inhibitors—Metformin, BAY 87-2243, IACS-010,759, MitoTam, complex II inhibitor—Lonidamine; general ECT inhibitor—VLX600) or inhibitors of the selected enzymes (PDH inhibitors—CPI-613 and dichloroacetate; GAPDH inhibitor—iodoacetate; PK inhibitor—lonidamine and lapachol; LDHA inhibitor—gossypol) (Hosseini et al. 2017; Missiroli et al. 2020). On the other hand, combination therapies involving metabolic effectors with an immune checkpoint blockade (ICB), chemotherapy, radiation and/or a diet may be even more effective than monotherapy, but only when the influence of each drug separately is known in detail (Bader et al. 2020).

Pyruvate dehydrogenase promotes the flux of carbohydrates into mitochondria and enhances aerobic oxidation of glucose. The known PDH cofactors are α-lipoic acid and ATP citrate lyase (ACL). Competitive treatment of B16–F10 melanoma cells with these compounds as well as with the hydroxycitrate inhibitor resulted in a significant cell growth inhibition (Fischer et al. 2018). Also, pyruvate dehydrogenase kinase is an important metabolic target, given that its inhibition shifts pyruvate metabolism from lactate (glycolysis) into the TCA cycle by increasing the activity of PDH. Dichloroacetate (DCA) is a pyruvate mimetic, which decreases the lactate production presumably through the inhibition of PDK activity. This was well documented by following the changes in ECAR and OCR of Melmet 5 cells (melanoma cell line investigated by the Seahorse analyzer). A DCA-induced reduction in ECAR and an increase in OCR was observed, which was accompanied by the elevation in the ATP level (Bettum et al. 2015). This DCA-dependent shift of metabolism from glycolysis to mitochondrial oxidation reverses mitochondrial dysfunction and reactivates mitochondria-dependent apoptosis in various tumor cells. The reduction of lactate accumulation also influences strongly the acidified TME (Li et al. 2016). Furthermore, DCA potentiates the effect of vemurafenib on only BRAF-mutated melanoma cells through a cooperative attenuation of energy production as it was identified by means of OCR/ECAR measurements. In this study, DCA was tested in the concentration range of 0.5–100 mM for up to 96 h and 50–100 nM VEM concentration on several melanoma cell lines. At the same time, there was no correlation between the cell response to only DCA and the expression levels of *PGC-1α* (Abildgaard et al. 2014). Due to the fact that melanoma cells resistant to vemurafenib maintained sensitivity to DCA, this approach suggests a possible combination therapy to overcome BRAF inhibitors resistance in patients with melanoma (Domingues et al. 2018). On the other hand, the key mediator of glycolysis in cancer cells is pyruvate kinase. Lapachol (LAP) is an analog of shikonin, which decreases the pyruvate kinase isozyme M2 (PKM2) activity at a micromolar range. Melanoma cells treated with lapachol showed a dose-dependent inhibition of glycolysis as well as a corresponding increase in oxygen consumption, observed by means of ECAR and OCR parameters, respectively. The blockade of glycolysis by lapachol in melanoma cells also led to the decreased ATP levels and inhibition of cell proliferation (Shankar Babu et al. 2018). Another modulator of PK is lonidamine (LND), which is able to sensitize human melanoma cells to doxorubicin (DOX) chemotherapy by acidifying and de-energizing the tumor. These results were obtained by LC–MS and Seahorse analysis (Nath et al. 2018).

The matter of interest is also the involvement of the glycolytic enzyme in mitochondria induced apoptosis. Mitochondrial permeability transition pore (mPTP), a voltage and Ca^2+^-dependent, cyclosporine A (CsA) and ROS sensitive channel in the inner mitochondrial membrane, is a key effector in the intrinsic mitochondrial apoptotic pathways and necrosis (Bonora et al. 2020). The mPTP is formed at the interface between two mitochondrial ATP synthase (F-ATP) dimers (Nesci 2018). Various adaptive mechanisms of tumor cells that desensitize the mPTP to Ca^2+^ and ROS, thereby playing an important role in the resistance of tumors to apoptosis, have already been described (Bernardi et al. 2015). The Ca^2+^- and ROS-dependent signaling pathways affecting transition of the F-ATP synthase from an energy-conserving to an energy-dissipating mechanism open new perspectives for therapeutic intervention aimed at the mPTP induction in cancer cells. Many used and potential chemotherapeutics that induce the mPTP, mainly by oxidative stress, are under investigation (Rasola and Bernardi 2014). Particularly interesting is isoform II of hexokinase (HK II), the first enzyme of glucose metabolism bound to the outer mitochondrial membrane at mitochondria‐endoplasmic reticulum contact sites that is predominantly expressed in malignant cells and has implications for tumor progression. The detachment of HK II from mitochondria induces mPTP opening and the subsequent apoptosis in several tumor cell models (Chiara et al. 2008; Ciscato et al. 2020). Substances triggering this process are being extensively researched (Bonora et al. 2020).

### Other reprogramming compounds

The endocannabinoid system (ECS), which consists of cannabinoid receptors, endogenous cannabinoids (endocannabinoids like N-arachidonylethanolamine, named anandamide) and enzymes responsible for the synthesis, transport and degradation of endocannabinoids, plays a dual role in tumorigenesis, the inhibition of tumor growth and metastatic spread (Moreno et al. 2018; Ramer et al. 2019). From a therapeutic point of view, the association of ECS with glucose and lipid metabolism is very interesting and some endocannabinoids are deeply involved in the maintenance of energy balance (Kim et al. 2011; Zaccagnino et al. 2011; Nava-Molina et al. 2020). Anandamide (AEA) has a confirmed action on the disturbance of the glucose uptake in skeletal muscle cells (Eckardt et al. 2009), thus one can assume that changes in the glucose distribution may affect general cell metabolism including the aberrant glycosylation of cells. This result may be observed due to the existing direct connection between glycolysis and the HBP pathway (Vasconcelos-dos-Santos et al. 2015). Moreover, AEA disturbs mitochondrial bioenergetics by the inhibition of oxidative phosphorylation in isolated mitochondria from rat liver (Zaccagnino et al. 2011).

Some antibiotics may also present a significant influence on cell metabolism by inducing mitochondrial dysfunction and oxidative damage in mammalian cells. Taking this into consideration, cell culture studies on metabolism should probably be done in an antibiotic free media (Elliott and Jiang 2019). An example is the class of aminoglycoside antibiotics, which consists mainly of gentamicin and geneticin. They show diverse usability against prokaryotic and eukaryotic cells, especially due to the fact that cell response depends on its metabolism (Sobiepanek et al. 2020c). The addition of gentamicin in cell culture media inhibits: the mitochondrial membrane potential gradient in the cells, the upregulation of *HIF1a* gene expression, the glycolytic enzymes and glucose transporters as well as the increase of lactate production, induction of mitochondrial superoxide and the increase in the DNA oxidative damage in the cells (Elliott and Jiang 2019). The addition of geneticin (G418) may cause the change of glucose flux from glycolysis towards either the TCA cycle or other biosynthetic pathways in various cell lines like baby hamster kidney (BHK) and Chinese hamster ovary (CHO) cells (Yallop and Svendsen 2001; Yallop et al. 2003).

On the other hand, there are various inhibitors of cell glycosylation, which can interrupt the biosynthesis of the complex type glycans. This generally decreases cell migration, adhesion and angiogenesis. Some of the well-known inhibitors of glycosidases are plant alkaloids like swainsonine (SW, α-mannosidase II and lysosomal α-mannosidase I inhibitor), deoxymannojirimycin (DMM, α-mannosidase I inhibitor), deoxynojirmycin (DNJ, glycosidase I), castanospermine (CS, glycosidase I and II inhibitor) (Powell 2001; Ciołczyk-Wierzbicka et al. 2004; Wojtowicz et al. 2012). Similarly interesting are sugar analogues like 2-deoxyglucose (2-DG) or 2-NBDG, which block glycotransferases and form dolichol-P-2-deoxyglucose that cannot be further extended. 2-DG has a further impact on the gene expression, protein phosphorylation of the signalling pathway, blocking the cell cycle progression, DNA repair and cell apoptosis (Wojtowicz et al. 2012; Lucena et al. 2016). Furthermore, 2-DG and 2-fluoro-deoxyglucose (2-FDG) are also the inhibitors of glycolysis, which compete for the HK binding (a key glycolytic enzyme) (Vasconcelos-dos-Santos et al. 2015; Missiroli et al. 2020).

## Conclusions

Melanoma is a highly heterogenous tumor, thus a combined therapy targeted at various metabolic pathways is needed to overcome the problem that many patients develop resistance to monotherapy. Novel factors able to remodel cell metabolism must be first analyzed separately to then be used in combined therapies. Various kinases and effectors of metabolism are currently the most promising drug target combination in therapy against melanoma. A detailed knowledge of each metabolic pathway is of great importance while analyzing the influence of new remodeling factors. This includes specific enzymes, transcription factors as well as proteins or glycoproteins. The most important pathways are glycolysis, TCA cycle, glutamine pathway, PPP pathway, HBP connected with the glycan biosynthesis pathway and in case of melanoma MAPK and mTOR pathways. For investigating various changes of specific markers and physical properties during melanoma development from nevus to metastases different types of analysis are highly recommended. These analyses include qPCR, Western blot/ELISA techniques, NMR, GC/LC–MS, Seahorse analysis as well as various label-free techniques (AFM, ITC, SPR, QCM/QCM-D).
